# P-155. Acute gastroenteritis and norovirus infection among high-risk adults: initial results from the ORION study

**DOI:** 10.1093/ofid/ofaf695.380

**Published:** 2026-01-11

**Authors:** Mark A Schmidt, Holly C Groom, Jennifer L Kuntz, Matthew T Slaughter, Claudia Steiner, Michael J Miller, Lisa Jackson, Jennifer K Meece, John F Dickerson, Michelle Blake, Christine Kim, Wen-Hsing Wu, Carly A Crocker, Brandon J Patterson, Meklit Workneh, Lee Quist, Katherine B Carlson

**Affiliations:** Center for Health Research, Kaiser Permanente Northwest, Portland, Oregon; Kaiser Permanente Center for Health Research, Portland, Oregon; Kaiser Permanente Center for Health Research, Portland, Oregon; Kaiser Permanente Northwest Center for Health Research, Portland, Oregon; Kaiser Permanente Institute for Health Research, Denver, Colorado; Kaiser Permanente Mid-Atlantic Permanente Research Institute, Washington, District of Columbia; Kaiser Permanente Washington Health Research Institute, Seattle, WA; Marshfield Clinic Research Institute, Marshfield, Wisconsin; Kaiser Permanente Center for Health Research, Portland, Oregon; Moderna, Inc., Cambridge, Massachusetts; Moderna, Inc., Cambridge, Massachusetts; Moderna, Inc., Cambridge, Massachusetts; Moderna, Inc., Cambridge, Massachusetts; Moderna, Inc., Cambridge, Massachusetts; Moderna, Inc, Cambridge, Massachusetts; Moderna, Inc., Cambridge, Massachusetts; Moderna, Cambridge, Massachusetts

## Abstract

**Background:**

The ongoing ‘Observational Research on the Impact and Outcomes of Norovirus’ (ORION) study is a prospective, community-based cohort study of acute gastroenteritis (AGE), and specifically norovirus (NoV) gastroenteritis (NGE), burden among adults with underlying medical conditions who may be at higher risk for severe NGE.

**Figure:**
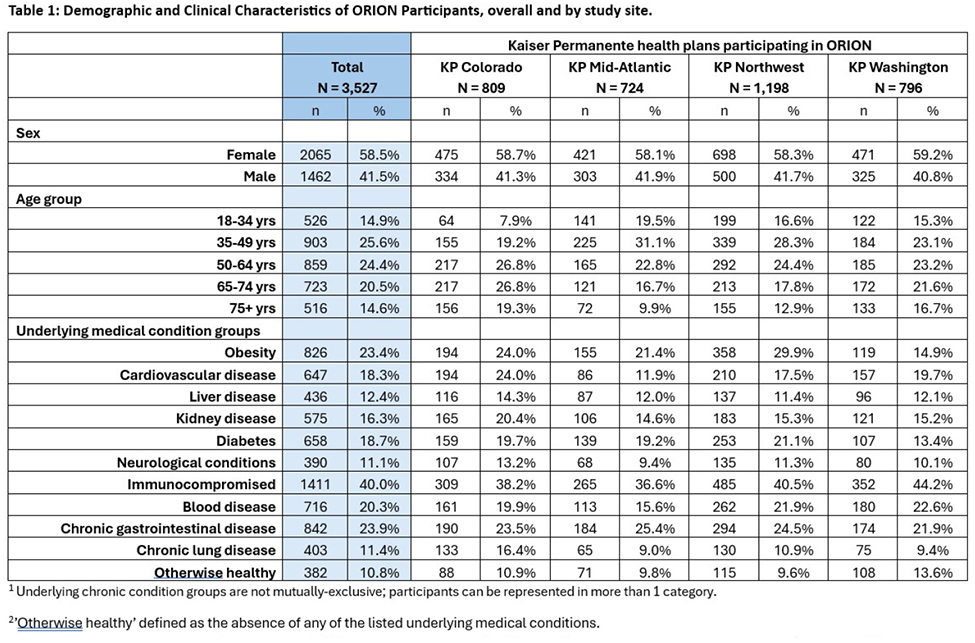

**Figure:**
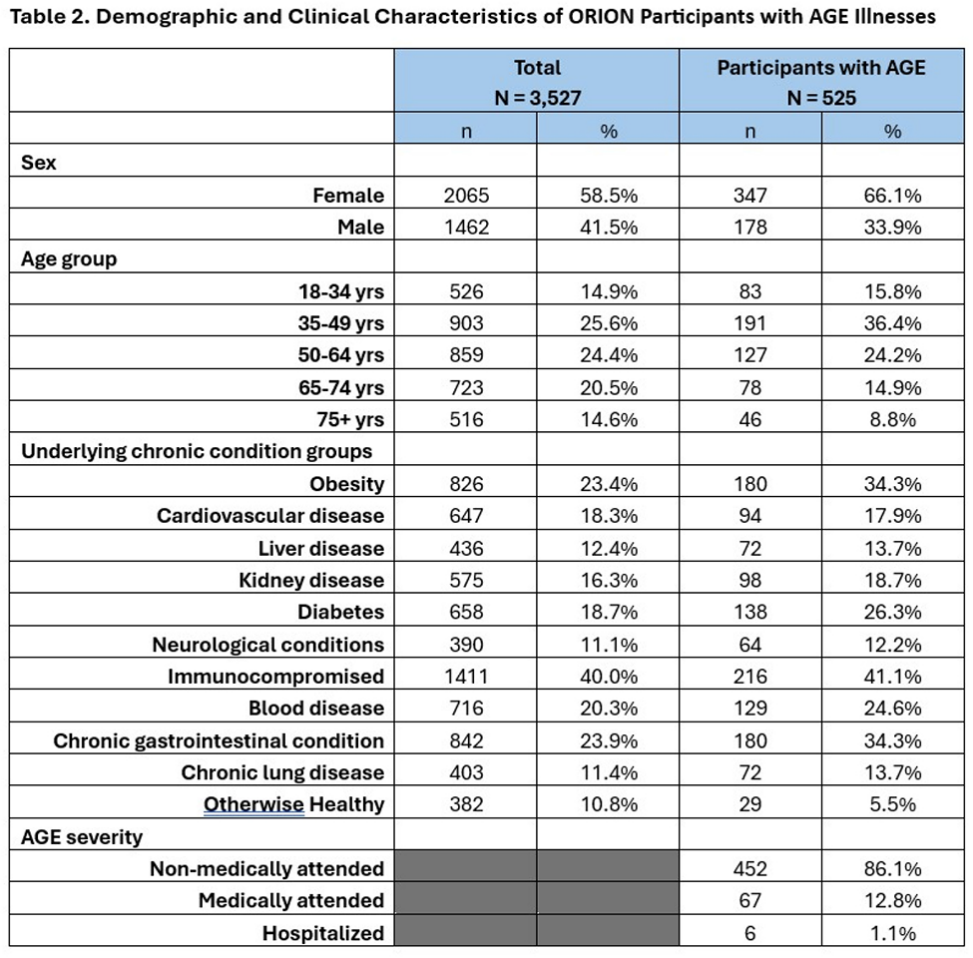

**Methods:**

From January–March 2025, we recruited adults (≥18 years) with pre-specified “high-risk” medical conditions [e.g., cardiovascular disease, gastrointestinal (GI) or immunocompromising (IC) conditions] and otherwise healthy controls from 4 Kaiser Permanente (KP) sites. Study participants report new onset of AGE weekly, defined as ≥1 vomiting episode and/or ≥3 diarrhea episodes in a 24-hour period. Participants with AGE: (1) complete surveys about illness severity, quality of life impacts, and exacerbation of underlying conditions and (2) self-collect stool specimens for multiplex GI pathogen testing. Here we report AGE episodes per 100 person-weeks (PW) and laboratory testing results through April 2025. Participant observation continues through December 2025.

**Figure:**
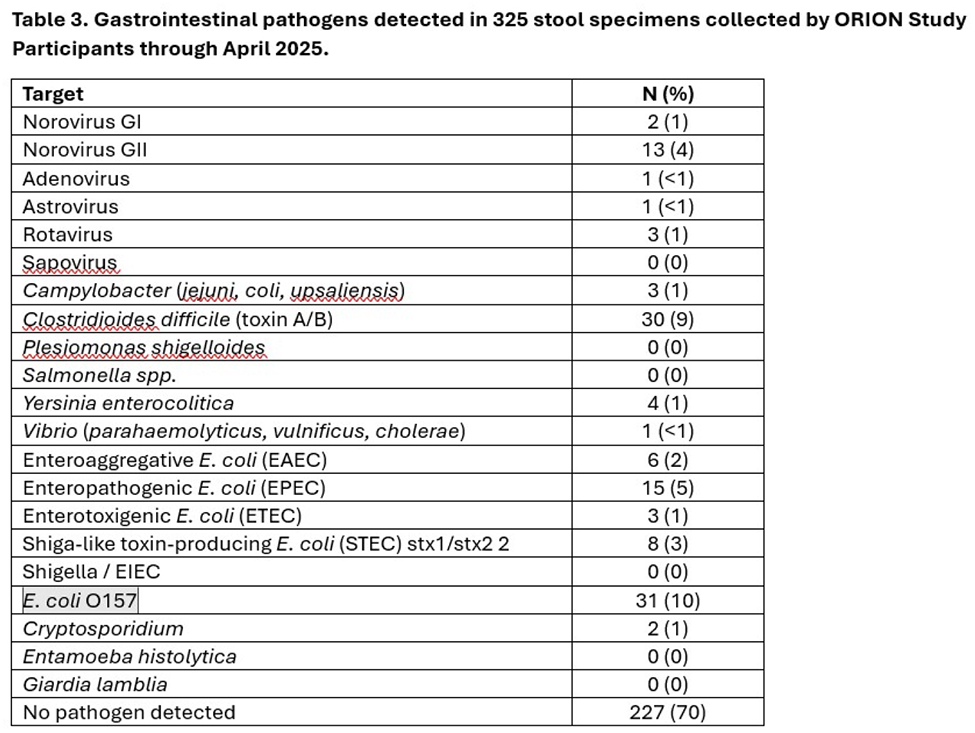

**Results:**

We enrolled 3,527 participants across the 4 sites, described in Table 1. Participants reported 525 AGE episodes over 15,329 PWs (incidence of 3.4/100 PW). AGE incidence peaked around 5-7/100 PWs in January/February and declined in March/April. Among participants with AGE (Table 2), most were female (66%) and either 35-49 years (36%) or 50-64 years (24%); 41% had IC conditions, 34% had GI conditions; 34% had obesity, and only 6% were otherwise healthy. Six AGE episodes involved hospitalization. In 325 stool specimens tested to date, 30% (n=98) had ≥1 pathogen detected and 5% (n=15) were NoV-positive (Table 3), with genotype GII comprising 87% of NoV-positive specimens.

**Conclusion:**

Burden of NGE in individuals with underlying medical conditions is not well understood and is often extrapolated from studies of medically attended AGE, which underestimate community disease burden. ORION will provide valuable AGE and NGE burden estimates from the community setting and allow for improved assessment of disease severity for adults with high-risk underlying medical conditions.

**Disclosures:**

Mark A. Schmidt, PhD, MPH, AstraZeneca: Grant/Research Support|HilleVax: Grant/Research Support|Janssen: Grant/Research Support|Moderna: Grant/Research Support Holly C. Groom, MPH, AstraZeneca: Grant/Research Support|Moderna: Grant/Research Support Jennifer L. Kuntz, MS, PhD, Astra Zeneca: Grant/Research Support|Moderna, Inc.: Grant/Research Support|Pfizer: Grant/Research Support Claudia Steiner, PhD, Moderna, Inc: Grant/Research Support Michael J. Miller, DrPH, American Journal of Health-System Pharmacy: Honoraria|Moderna: Grant/Research Support|Pfizer: Grant/Research Support|The American Society of Health-System Pharmacists, Inc: Grant/Research Support Lisa Jackson, MD, MPH, Moderna: Grant/Research Support Jennifer K. Meece, PhD, CSL Seqirus: Grant/Research Support|GSK: Grant/Research Support|ModernaTX: Grant/Research Support John F. Dickerson, PhD, AstraZeneca: Grant/Research Support|HilleVax: Grant/Research Support|Moderna: Grant/Research Support Michelle Blake, PhD, MSc, HBSc, Moderna: Employment|Moderna: Stocks/Bonds (Public Company) Christine Kim, PhD, MSPH, Director, Moderna, Inc.: Employee|Director, Moderna, Inc.: Stocks/Bonds (Public Company) Wen-Hsing Wu, MS, Moderna: Stocks/Bonds (Public Company) Carly A. Crocker, BS, Moderna: Employment|Moderna: Stocks/Bonds (Public Company) Brandon J. Patterson, PharmD, PhD, Moderna: Employment|Moderna: Stocks/Bonds (Private Company) Meklit Workneh, MD, MPH, Moderna: Stocks/Bonds (Public Company) Lee Quist, DO, MBA, Moderna: Stocks/Bonds (Public Company) Katherine B. Carlson, PhD, MPH, Moderna: Employee|Moderna: Stocks/Bonds (Private Company)

